# Provincial and territorial congenital anomalies surveillance: a summary of surveillance programs across Canada

**DOI:** 10.24095/hpcdp.44.5.04

**Published:** 2024-05

**Authors:** Tanya Bedard, Yonabeth Nava de Escalante, Cora Cole, Kitty Dang, Maya Jeyaraman, Kathryn Johnston, Qun Miao, Lauren Rickert, Chantal Nelson

**Affiliations:** 1 Alberta Congenital Anomalies Surveillance System, Alberta Health Services, Calgary, Alberta, Canada; 2 Office of the Provincial Health Officer, BC Ministry of Health, Victoria, British Columbia, Canada; 3 Nova Scotia Reproductive Care Program, Halifax, Nova Scotia, Canada; 4 Perinatal Surveillance Health PEI, Charlottetown, Prince Edward Island, Canada; 5 Government of the Northwest Territories, Department of Health and Social Services, Yellowknife, Northwest Territories, Canada; 6 Manitoba Health, Winnipeg, Manitoba, Canada; 7 New Brunswick Perinatal Health Program, Moncton, New Brunswick, Canada; 8 BORN Ontario, Ottawa, Ontario, Canada; 9 Newfoundland and Labrador Health Services, St. John’s, Newfoundland and Labrador, Canada; 10 Centre for Surveillance and Applied Research, Public Health Agency of Canada, Ottawa, Ontario, Canada

## Abstract

The Canadian Congenital Anomalies Surveillance Network was established in 2002 to address gaps in the national surveillance of congenital anomalies (CAs) and support the sustainability of high-quality, population-based, CA surveillance systems within provinces and territories. This paper highlights the methodologies of each local CA surveillance system, noting similarities and variabilities between each system, to contribute to enhanced national CA surveillance efforts.

**Highlights**

- The Canadian Congenital Anomalies Surveillance Network was established in 2002 under the umbrella of the Canadian Perinatal Surveillance System to support highquality, population-based congenital anomalies surveillance systems in Canada. Each local congenital anomalies surveillance system covers diverse populations and geography, operates under different structures and has varying program maturity.
- Engagement of every jurisdiction is essential for sustaining local and national CA surveillance.
- Provincial and territorial CA surveillance systems are uniquely positioned to support public health priorities.

## Introduction

Congenital anomalies (CAs) are the leading cause of infant deaths in Canada^1^ and one of the most frequent causes worldwide.^2^ Congenital anomalies surveillance systems were established globally after the thalidomide tragedy, including in Canada, with the national Canadian Congenital Anomalies Surveillance System (CCASS).^3^,^4^

However, gaps exist in the CCASS data and there are opportunities to address the limitations.^4^ Historically, administrative health data ascertained from the Canadian Institute for Health Information (CIHI) Discharge Abstract Database (DAD) have been exclusively used for CCASS.^3^,^4^ The CIHI-DAD comprises hospital discharge data for all provinces and territories, except for Quebec, and is used to identify cases with CAs.^5^ The Public Health Agency of Canada (PHAC) developed linkage methodologies to follow up infant admissions that occur up to one year of age; however, this is not sufficient for complete ascertainment of CAs in Canada, as data may be incomplete.^6^ There are limitations in the CIHI-DAD for stillbirths, elective termination of pregnancies for fetal anomalies (ETOPFA), environmental exposures, and individual risk factors, all of which also impact the completeness of the CCASS.

The Canadian Congenital Anomalies Surveillance Network was established in 2002, within the Canadian Perinatal Surveillance System. The goal of this network is to enhance CA surveillance data. Members include clinicians, academics and public health professionals from across Canada. 

The *Action Plan to Protect Human Health from Environmental Contaminants*, announced by the Government of Canada in 2008, is a federal initiative designed to protect the health of Canadians from harmful environmental contaminants.^6^ Under this action plan, PHAC, with support from the Canadian Congenital Anomalies Surveillance Network, works with provinces and territories to establish or enhance local CA surveillance systems to improve CA surveillance in Canada. This will address gaps in national CA surveillance, since local data are more complete and accurate.^7^ The objective of this paper is to provide an overview of each provincial and territorial CA surveillance system that supports enhanced local and national surveillance activities.

## Methods

The Canadian Congenital Anomalies Surveillance Network Data Publication Working Group conducted a survey based on the National Birth Defects Prevention Network’s State Birth Defects Surveillance Program Directory.^8^ This survey was modified to ascertain program level details across jurisdictions as shown in Table 1.

**Table 1:** Overview of provincial and territorial congenital anomalies registry and surveillance programs in Canada

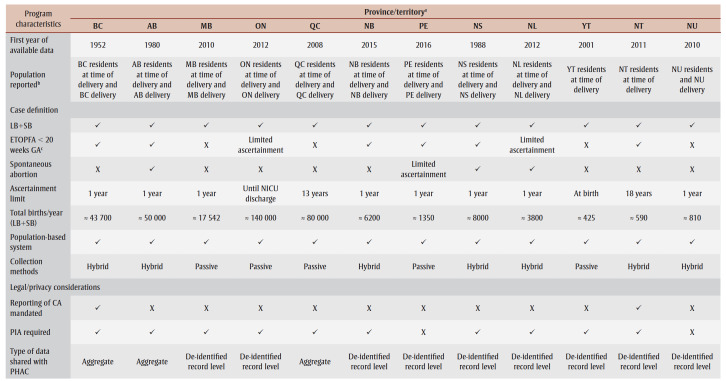 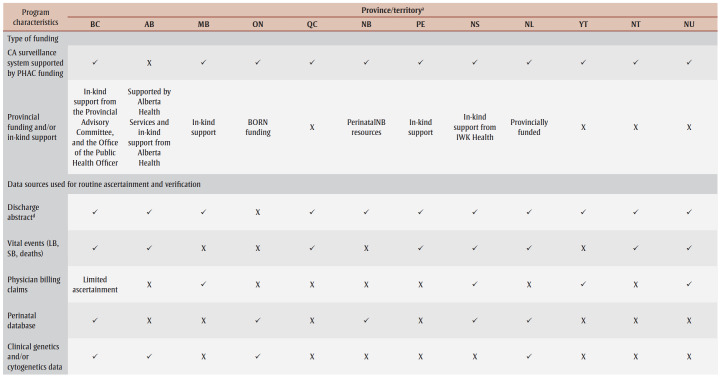 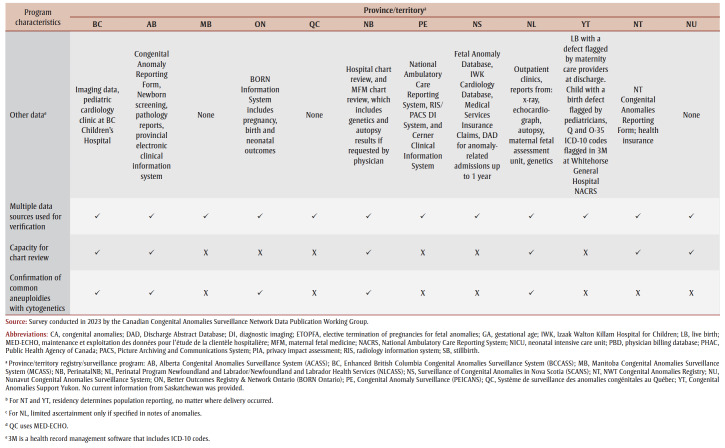

Every province and territory (British Columbia [BC], Alberta [AB], Saskatchewan [SK], Manitoba [MB], Ontario [ON], Quebec [QC], New Brunswick [NB], Prince Edward Island [PE], Nova Scotia [NS], Newfoundland and Labrador [NL], Yukon [YT], Northwest Territories [NT] and Nunavut [NU]) had opportunities to respond to the survey via email correspondence or through one-on-one interviews in May 2023. Only one representative from each jurisdiction was eligible to respond to the survey, with completion implying consent. Qualitative data were analyzed using thematic analysis and constant comparison methodology.^9^

Health Research Ethics Board (HREB) review and approval were not required, as it was considered a quality assurance project and fell within the scope of CA surveillance practice, and no identifiable registry data were accessed.^10^

## Results

Representatives from nine provinces and three territories completed the survey, for a completion rate of 92%. The results of the survey provide an overview of the current state and activities of CA surveillance for each jurisdiction (Table 1).

All local systems report on births occurring within their mother’s place of residence. However, two have the capacity to report on out-of-province births. All include live births and stillbirths in their case definition, with half routinely ascertaining early ETOPFAs (i.e. delivered < 20 weeks gestational age). Two additional provinces (ON and NL) have limited ascertainment for early ETOPFAs. One year after delivery is the case ascertainment limit for most jurisdictions, except for NT (18 years), QC (13 years), ON (discharge from a neonatal intensive care unit) and YT (at birth).

Every system is population-based, with seven using a hybrid method of ascertainment. For instance, they passively receive case notifications and actively ascertain cases using additional data sources such as health records to contribute to completeness and accuracy of CAs within their jurisdiction. The remaining five rely on passive methods. All jurisdictions use multiple data sources, while nine out of 12 verify cases using additional clinical data (e.g. pediatric cardiology).

The reporting of CAs is mandated with supporting legislation in only two jurisdictions (BC and NT). While all programs have undergone privacy and/or ethical reviews, 10 out of 12 have completed a full privacy impact assessment. Three-quarters (9/12) share de-identified record–level data with PHAC, while the remaining share aggregate data. Funding from PHAC helps support 11 out of 12 systems, and 8 out of 12 receive provincial/territorial and/or in-kind supports.

## Discussion

The Public Health Agency of Canada, with the support of the Canadian Congenital Anomalies Surveillance Network, has actively engaged each province and territory to establish or enhance local CA surveillance to strengthen national surveillance efforts under the *Action Plan to Protect Human Health from Environmental Contaminants*.^6^ Each jurisdiction began at a different stage, with some already operating CA surveillance systems, and others needing to be established or revamped. As highlighted in Table 1, each program is unique and incorporates local diverse populations and geography. 

The ascertainment and reporting of out-of-province cases are substantially limited. Provinces and territories report a wide range in the percentage of deliveries that occur outside of their jurisdiction—anywhere fromless than1% up to 75% (data not shown). Due to existing local case definitions and the lack of interjurisdictional data sharing agreements, births or deliveries outside of the mother’s place of residence with CAs are missed. Thus, it is difficult to report on the true burden of CAs. This supports the need for inter-provincial and -territorial data sharing agreements to enhance local surveillance efforts. 

The ascertainment of ETOPFAs significantly improves data quality (i.e. completeness), particularly when those less than 20 weeks gestational age are included. Many pregnancies with lethal or severe anomalies (e.g. anencephaly) are terminated early and are not included in most passive systems.^11^,^12^ Two-thirds of local systems include at least limited data for early ETOPFAs, which provides more accurate estimates, particularly for more severe CAs, compared to those that do not include early ETOPFAs. It is also important to distinguish spontaneous stillbirths from ETOPFA at or greater than20 weeks gestational age. The current definition of stillbirths needs updating to reflect this key distinction, as it has a significant impact on CA and stillbirth surveillance efforts.^13^ Nine jurisdictions have the capacity to distinguish spontaneous stillbirths from ETOPFA at or greater than 20 weeks gestational age (data not shown).

All local systems use multiple data sources, including some with clinical data, to contribute to the verification of cases. The capacity to use both passive and active components for CA surveillance, and to verify data using multiple data sources, increases confidence in data quality. This differs from CCASS, which is a passive system that has historically primarily used one health administrative data source for reporting6 and research.^14^,^15^ Administrative health data are not collected for the purpose of CA surveillance; thus, there are data quality limitations. A previous comparison of CCASS with a provincial CA surveillance system showed that although there was satisfactory agreement between the two systems for some major anomalies, there was often an overestimation of anomalies in CCASS due to a lack of validation and issues with classification and coding.^16^ This limitation was also reported when comparing administrative health datasets with dedicated local CA surveillance systems.^17^,^18^ Although hybrid case ascertainment is more resource intensive, it results in more complete and accurate data. This is particularly relevant for rare anomalies and jurisdictions with lower population numbers, as the misclassification and coding of cases can significantly impact prevalence.

Many local programs collaborate with and are supported by experts in a variety of specialties (e.g. maternal fetal medicine, genetics) and their provincial or territorial advisory group (where they exist). Local CA surveillance systems are better positioned to respond to cluster investigations, program planning and resource allocation and to support local interests and needs than a national level system. 

Dedicated PHAC funding has provided opportunities for most local systems to establish or enhance CA surveillance within their jurisdiction and contribute to national CA surveillance activities. Funding from health authorities, provincial and territorial governments, and in-kind supports also contribute to local CA surveillance activities. For some programs, dedicated provincial or territorial funding is essential to support operations and sustainability over time.

***Strengths and limitations***

Almost all provinces and territories completed the survey and reflect the status of CA surveillance across Canada. Representatives from Saskatchewan were invited to participate; however, they were not able to provide any current information. Engagement with provinces and territories, the Canadian Congenital Anomalies Surveillance Network and PHAC contribute to a strengthened CCASS.

Addressing the diversity of each province and territory with a relatively short survey was challenging, and highlights the need for continued engagement and standardization across the country.

## Conclusion

The engagement and investment to date from PHAC, provincial and territorial governments, and health authorities have been essential to sustain local and national CA surveillance, as were the efforts and dedication of the Congenital Anomalies Surveillance Network. While national CA surveillance can be reliable in smaller countries, such reliability and accuracy are challenging to achieve in geographically larger countries, highlighting the need for local systems to strengthen national surveillance in Canada.^3^,^7^ To further enhance CA surveillance in Canada, interjurisdictional data sharing agreements are required. 

## Acknowledgements

We would like to thank all members of the Canadian Congenital Anomalies Surveillance Network for their continued commitment to the development and maintenance of high-quality, population-based surveillance systems of congenital anomalies that will provide information to improve the health of Canadian children and their families.

## Conflicts of interest

This manuscript was supported by the Public Health Agency of Canada (PHAC; to YN); by BORN Ontario (to QM); and by the BC Ministry of Health (to YN). PHAC provides funding to LR and MJ to attend meetings and travel. LR is supported by electronic equipment and office space provided by Newfoundland and Labrador Health Services and by salary funding provided to Newfoundland and Labrador Health Services from PHAC, in-kind with Newfoundland and Labrador Health Services. MJ received a grant from the Canadian Institutes of Health Research in 2020 for an unrelated research project. CC is supported by contract funding provided to the IWK Health Centre from PHAC for work related to Nova Scotia and in-kind contributions to represent Health PEI. CN is a contract lecturer at Lakehead University and Carleton University.

## Authors’ contributions and statement

CN, CC, QM, KJ, KD, LR, MJ, TB, YN—conceptualization. 

CC, QM, KJ, KD, LR, MJ, TB, YN—analysis. 

CN, CC, QM, KJ, KD, LR, MJ, TB, YN—writing—original draft.

The content and views expressed in this article are those of the authors and do not necessarily reflect those of local health agencies, provincial and territorial governments, and the Government of Canada.
